# MZF1 in the Dorsal Root Ganglia Contributes to the Development and Maintenance of Neuropathic Pain via Regulation of TRPV1

**DOI:** 10.1155/2019/2782417

**Published:** 2019-09-08

**Authors:** Fei Xing, Hanwen Gu, Qin Niu, Xiaochong Fan, Zhongyu Wang, Jingjing Yuan, Zhisong Li, Ji-Tian Xu, Wei Zhang

**Affiliations:** ^1^Department of Anesthesiology, The First Affiliated Hospital, Zhengzhou University, 1 Jianshe East Road, Zhengzhou 450052, China; ^2^Department of Physiology and Neurobiology, School of Basic Medical Sciences, Zhengzhou University, 100 Science Avenue, Zhengzhou 450001, China; ^3^Neuroscience Research Institute, Zhengzhou University Academy of Medical Sciences, 100 Science Avenue, Zhengzhou 450001, China

## Abstract

Previous studies have demonstrated that myeloid zinc finger 1 (MZF1) in the dorsal root ganglion (DRG) participates in neuropathic pain induced by chronic-constriction injury (CCI) via regulation of voltage-gated K^+^ channels (Kv). Emerging evidence indicates that transient receptor potential vanilloid 1 (TRPV1) is involved in the development and maintenance of neuropathic pain. Although it is known that the transcription of TRPV1 is regulated by Kruppel-like zinc-finger transcription factor 7 (Klf7)—and that the structure of TRPV1 is similar to that of Kv—few studies have systematically investigated the relationship between MZF1 and TRPV1 in neuropathic pain. In the present study, we demonstrated that CCI induced an increase in MZF1 and TRPV1 in lumbar-level 4/5 (L4/5) DRGs at 3 days post-CCI and that this increase was persistent until at least 14 days post-CCI. DRG microinjection of rAAV5-MZF1 into the DRGs of naïve rats resulted in a decrease in paw-withdrawal threshold (PWT) and paw-withdrawal latency (PWL) compared with that of the rAAV5-EGFP group, which started at four weeks and lasted until at least eight weeks after microinjection. Additionally, prior microinjection of MZF1 siRNA clearly ameliorated CCI-induced reduction in PWT and PWL at 3 days post-CCI and lasted until at least 7 days post-CCI. Correspondingly, microinjection of MZF1 siRNA subsequent to CCI alleviated the established mechanical allodynia and thermal hyperalgesia induced by CCI, which occurred at 3 days postinjection and lasted until at least 10 days postinjection. Microinjection of rAAV5-MZF1 increased the expression of TRPV1 in DRGs. Microinjection of MZF1 siRNA diminished the CCI-induced increase of TRPV1, but not P2X7R, in DRGs. These findings suggest that MZF1 may contribute to neuropathic pain via regulation of TRPV1 expression in DRGs.

## 1. Introduction

Neuropathic pain has become a major public health problem. Although there have been improvements in understanding its pathophysiological mechanisms, optimal treatment of neuropathic pain has continued to be a clinical challenge for physicians [1]. It is generally believed that peripheral sensitization and central sensitization play an important role in the development and maintenance of neuropathic pain [2]. Dorsal root ganglion (DRG) neurons serve as a bridge between the internal and external environments and the spinal cord link between the transmission of peripheral sensitization and central sensitization. The abnormal activity of primary neurons in DRGs is related to neuropathic pain induced by nerve injury, and these neurons enhance peripheral sensitization and affect expression of pain-related receptors, enzymes, and ion channels in DRGs [3, 4].

Myeloid zinc finger 1 (MZF1) is a Cys2His2 (C2H2) zinc-finger transcription factor, belonging to the Kruppel family, which activates and inhibits the process of transcription to participate in cell differentiation, hematopoietic and nonhematopoietic cell migration, and procreation [5, 6]. A great deal of research has been conducted to prove the role of MZF1 in cancer as a gene repressor or activator [7]. A previous study found that MZF1 is involved in the regulation of the expression of voltage-gated K+ channel (Kv) genes in rats undergoing chronic-constriction injury (CCI) and that MZF1 enhances the excitability and frequency of ectopic discharge of DRG neurons [8, 9], which results in peripheral sensitization. Although previous research has demonstrated the importance of MZF1 in neuropathic pain, few studies have observed the mechanisms underlying induction of neuropathic pain.

TRPV1 as a nonselective cationic channel is a member of the transient receptor potential family and is widely distributed in medium and small neurons [10]. TRPV1 integrates multiple kinds of nociceptive stimuli and regulates the downstream function of multiple signaling pathways. TRPV1 constantly restores and enhances its own activity, which can result in the continuous abnormal activation of neurons and, thus, trigger the occurrence and maintenance of chronic pain [11]. It has been reported that a novel TRPV1 antagonist (V116517) had a potent antihyperalgesic effect in a randomized, double-blind, positive-controlled, 3-way crossover human experimental pain study [12]. The expression of TRPV1 is regulated by zinc-finger transcription factors and its structure is similar to that of Kvs, but whether MZF1 participates in the regulation of transcription and expression of TRPV1 to contribute to the development and maintenance of neuropathic pain requires further investigation.

In the present study, we examined whether the mRNA and protein expression of MZF1 and TRPV1 increased in lumbar-level 4/5 (L4/5) DRGs following CCI. We observed the distribution of MZF1 and TRPV1 in the L4/5 DRG on day 7 after CCI. Next, we used rAAV5-MZF1 to examine whether overexpression of MZF1 mimicked CCI-induced neuropathic pain and used MZF1 siRNA and TRPV1 siRNA to examine the role of MZF1 and TRPV1 in the development and maintenance of neuropathic pain. Finally, we examined whether blocking DRG MZF1 abolished any CCI-induced increase in TRPV1 in DRGs.

## 2. Materials and Methods

### 2.1. Preparation of Animals

Male Sprague Dawley rats weighing 200–300 g (purchased from the Laboratory Animal Center of Zhengzhou University, Zhengzhou, Henan Province, China) were used in this experiment. The rats were free to eat and drink in their respective cages. The indoor temperature was maintained at 23 ± 2°C, the relative humidity was 60–70%, and we employed the natural rhythm of the 12–12 h light-dark cycle. All animal experimental procedures were approved by the Institutional Animal Care and Use Committee of Zhengzhou University and were carried out in accordance with the guidelines of the National Institutes of Health on animal care.

### 2.2. Chronic-Constriction Injury Model

Our CCI model followed procedures that have been described previously [13, 14]. Briefly, the rats were placed under anesthesia with sevoflurane (2–3%) vaporized through a nose cone and were scrubbed with 10% povidone-iodine three times. An incision was made on the lateral surface of the midthigh, the biceps femoris muscle was blunt-dissected, and the sciatic nerve was exposed and loosely ligated with 5-0 silk at four sites with an interval of 1 mm. For the sham group, all procedures were identical to those in the CCI group, except that there was no nerve ligation. The wound was sutured with 3-0 silk.

### 2.3. Behavioral Tests

Pain-related behavioral tests were performed according to our previously described methods [15, 16]. All animals were acclimated to the testing environment two days before baseline testing. Von Frey hairs were used to stimulate the plantar surface of the hind paw to evaluate paw-withdrawal threshold (PWT), and 50% PWT was determined using the up-down method [17]. Briefly, a series of Von Frey hairs with ascending stiffness (0.41, 0.70, 1.20, 2.04, 3.63, 5.50, 8.51, and 15.14 g) was used to stimulate the plantar surface of the hind paw, starting from the 2.0 g filament stimulus. The paw-withdrawal latency (PWL) was measured by the plantar test (7370, Ugo Basile, Comeria, Italy) and used the method of Hargreaves et al. [18]. Briefly, a radiant heat source beneath a glass floor was applied at the plantar surface of the hind paw. Each rat was tested three times with 5 min intervals between consecutive, alternating tests. The average of the three measurements was taken as the result per test.

### 2.4. DRG Microinjections

DRG microinjections were performed in accordance with the previous method described by Sun et al. [19]. Briefly, rats were anesthetized with sevoflurane (2–3%), and the lower lumbar-back region was scrubbed with 10% povidone-iodine three times. An incision of the median back was made, and the lumbar articular process was removed. The exposed L4/5 DRG was injected with a viral solution or siRNA-mixed solution through a glass micropipette connected to a Hamilton syringe. The pipette remained in the same position 10 min after injection. Rats with paresis or other abnormalities after surgery were excluded from further experiments.

### 2.5. Western Blotting

Western blotting was carried out following a procedure that we have described previously [20, 21]. The L4/5 DRGs were collected and quickly stored in liquid nitrogen. The samples were homogenized with an ice-cold lysis buffer (10 mM Tris, 5 mM EGTA, 0.5% Triton X-100, 2 mM benzamidine, 0.1 mM PMSF, 40 mM leupeptin, 150 mM NaCl, and 1% phosphatase inhibitor cocktails 2 and 3). The crude homogenate was centrifuged at 4°C for 15 min at 3000 r/min, and the supernatant was collected. The concentration of protein was measured according to the Bradford method. Samples were denatured at 99°C for 5 min and electrophoresed using 10% SDS-polyacrylamide gels. Then, the proteins were electrophoretically transferred onto PVDF membranes (Millipore, IPVH85R). The membranes were blocked with 3% nonfat milk in Tris-buffered saline containing 0.1% Tween-20 for 1 h and then incubated with primary antibodies overnight at 4°C. The primary antibodies used were as follows: rabbit anti-MZF1 (1 : 1,000, ABclonal A10356), rabbit anti-TRPV1 (1 : 1,000, Abcam, ab6166), rabbit anti-P2X7R (1 : 2,000, ABclonal, A10511), and mouse anti-*β*-actin (1 : 10,000; Sigma). The membranes were detected by horseradish peroxidase-conjugated anti-mouse or anti-rabbit secondary antibodies and visualized by chemiluminescent reagents from the Immobilon Western Chemiluminescent HRP Substrate (Millipore, WBKLS0100) and exposed to film. The intensity of Western blotting was quantified with densitometry. The blot density from control groups was set as 100%. The relative-density values from the other groups were determined by dividing the optical density values from these groups by the control value after each was normalized to its corresponding *β*-actin.

### 2.6. Immunohistochemistry

Immunohistochemistry was performed according to a method described previously [22]. Briefly, after defined survival times, sham and CCI rats were terminally anesthetized and perfused through the ascending aorta with saline, followed by 4% paraformaldehyde in a 0.1 M phosphate buffer. L4/5 DRGs were removed and postfixed for 3 h in the same fixative and were then placed in 30% sucrose phosphate-buffered saline for two nights. The transverse DRG sections (16 *μ*m) were cut on a cryostat (LEICA CM1900) and prepared for immunostaining. Blocking sections with 5% goat serum in 0.3% Triton X-100 for 1 h at 37°C were incubated with primary antibody overnight at 4°C after washing with phosphate-buffered saline. The primary antibodies used were as follows: rabbit anti-MZF1 (1 : 100, ABclonal A10356) and rabbit anti-TRPV1 (1 : 200, Abcam, ab6166). For double-immunofluorescent staining, all the sections (except for IB4-treated DRG sections, which were only incubated with Cy3-conjugated secondary antibody) were treated by a mixture of goat anti-mouse FITC- (1 : 200, Jackson ImmunoResearch, Amish, PA) and goat anti-rabbit Cy3-conjugated secondary antibodies (1 : 400, Jackson ImmunoResearch) for 1 h at 37°C. Finally, sections were mounted onto glass slides and visualized using an Olympus IX73 (Olympus Optical, Tokyo, Japan) fluorescent microscope, and images were captured with a CCD-spot camera.

### 2.7. RNA Extraction and Quantitative Real-Time PCR

Quantitative real-time PCR (qRT-PCR) was performed following a method described previously [23]. Animals were euthanized by decapitation, and the L4-L5 DRGs were harvested and placed in TRIzol. The total RNA was extracted by the TRIzol method. Total RNA was then reverse-transcribed using an oligo-dT primer and PrimeScript II RTase (TaKaRa). Each sample was run in triplicate in a 20 *μ*L reaction with 10 *μ*M forward and reverse primers, 10 *μ*L of SYBR Green qPCR Super Mix (Invitrogen), and 25 ng of cDNA. Reactions were performed in the Applied Biosystems 7500 Fast Real-Time PCR System. GAPDH was used as an internal control to normalize samples. The relative ratio of ipsilateral-side mRNA levels to contralateral-side mRNA levels was quantified by the 2^−*ΔΔ*CT^ method. The rat-specific primer sequences of the examined mRNA for qRT-PCR are listed in Table 1.

### 2.8. Statistical Analysis

Statistical tests were performed with SPSS 10.0 (SPSS Inc., USA) and SigmaStat (Systat, San Jose, CA). All data are expressed as mean ± SEM. For behavioral analyses, all group and between-group comparisons were performed using a two-way ANOVA followed by Tukey's *post hoc* test, and two groups at the same time points were tested by Student's *t*-tests. For qRT-PCR, Western blotting, and immunohistochemistry, the data among different groups were analyzed using one-way ANOVAs followed by Tukey's *post hoc* tests, or Student's *t*-tests if only two groups were compared. We considered a criterion of *P* < 0.05 as statistically significant.

## 3. Results

### 3.1. CCI Increases the Expression of MZF1 and TRPV1 in DRGs

**Figure 1:**
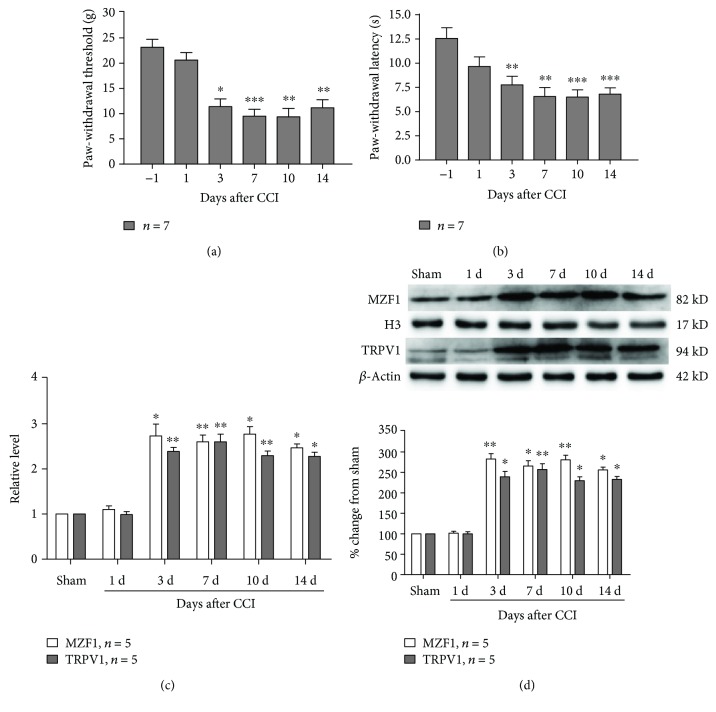
Chronic-constriction injury (CCI) induces mechanical allodynia and thermal hyperalgesia and increases MZF1 and TRPV1 levels in L4/5 DRGs. (a, b) Behavioral data showing the reduction of paw-withdrawal threshold (PWT) (a) and paw-withdrawal latency (PWL) (b) following CCI. ^∗^*P* < 0.05, ^∗∗^*P* < 0.01, and ^∗∗∗^*P* < 0.001 vs. baseline value (two-way ANOVA). (c) qRT-PCR data showing an increase in mRNA expression of MZF1 and TRPV1 in L4/5 DRGs following CCI. ^∗^*P* < 0.05 and ^∗∗^*P* < 0.01 vs. the sham group (one-way ANOVA). (d) Western blotting data showing an increase in protein expression of MZF1 and TRPV1 in L4/5 DRGs following CCI. ^∗^*P* < 0.05 and ^∗∗^*P* < 0.01 vs. the sham group (one-way ANOVA).

As previously demonstrated, CCI resulted in a rapid decrease of PWT (compared with that of the preoperative group, 3 d, *P* < 0.05; 7 d, *P* < 0.001; 10 d, *P* < 0.01; and 14 d, *P* < 0.01; one-way ANOVA, Figure 1(a)) and PWL (compared with that of the preoperative group, 3 d, *P* < 0.01; 7 d, *P* < 0.01; 10 d, *P* < 0.001; and 14 d, *P* < 0.001; one-way ANOVA, Figure 1(b)), which started at 3 d post-CCI and persisted for at least 14 d post-CCI. Additionally, qRT-PCR data showed that CCI resulted in an increase in the mRNA expression of MZF1 (compared with that of the sham group, 3 d, *P* < 0.05; 7 d, *P* < 0.01; 10 d, *P* < 0.05; and 14 d, *P* < 0.05; one-way ANOVA, Figure 1(c)) and TRPV1 (compared with that of the sham group, 3 d, *P* < 0.01; 7 d, *P* < 0.01; 10 d, *P* < 0.01; and 14 d, *P* < 0.05; one-way ANOVA, Figure 1(c)) in L4/5 DRGs at 3 d post-CCI, and these increases persisted for at least 14 d post-CCI. In order to corroborate the above results, we employed Western blotting to examine the protein alterations of MZF1 and TRPV1 in L4/5 DRGs after CCI. The results of Western blotting also showed that the protein expression of MZF1 (compared with that of the sham group, 3 d, *P* < 0.01; 7 d, *P* < 0.05; 10 d, *P* < 0.01; and 14 d, *P* < 0.05; one-way ANOVA, Figure 1(d)) and TRPV1 (compared with that of the sham group, 3 d, *P* < 0.05; 7 d, *P* < 0.01; 10 d, *P* < 0.05; and 14 d, *P* < 0.05; one-way ANOVA, Figure 1(d)) was time-dependently increased in the ipsilateral L4/5 DRG following CCI, for which the increase was detectable at 3 d post-CCI and remained for 14 d post-CCI.

**Figure 2:**
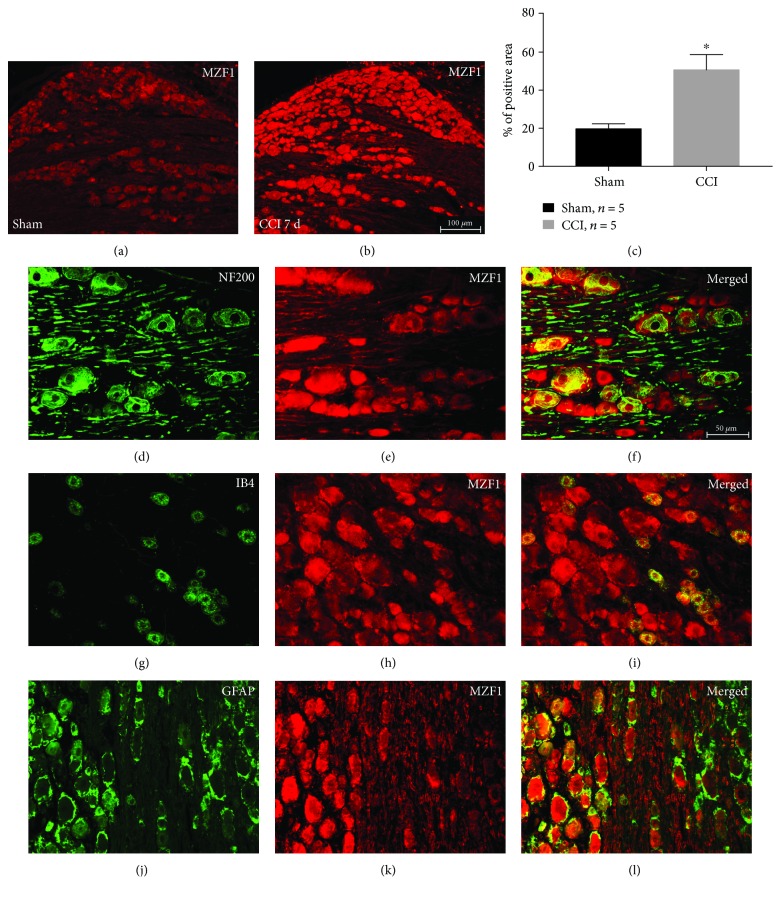
MZF1 immunoreactivity (IR) in DRGs increases after CCI. (a, b) Representative images showing MZF1 IR in DRGs for sham-surgery control (a) and at 7 days post-CCI (b). Scale bar for (a) and (b): 100 *μ*m. (c) Quantification of MZF1-positive area in DRGs. ^∗^*P* < 0.05 vs. the sham group Student's *t*-test. (d–l) Double-labeled immunofluorescent images showing that MZF1 (red: (e), (h), and (k)) colocalized with the A-type neuron marker NF200 (green: (d)), C-type neuron marker IB4 (green: (g)), and satellite glial-cell marker GFAP (green: (j)). The pairs of images are merged in (f), (i), and (l). Scale bar for (d)–(l): 50 *μ*m.

**Figure 3:**
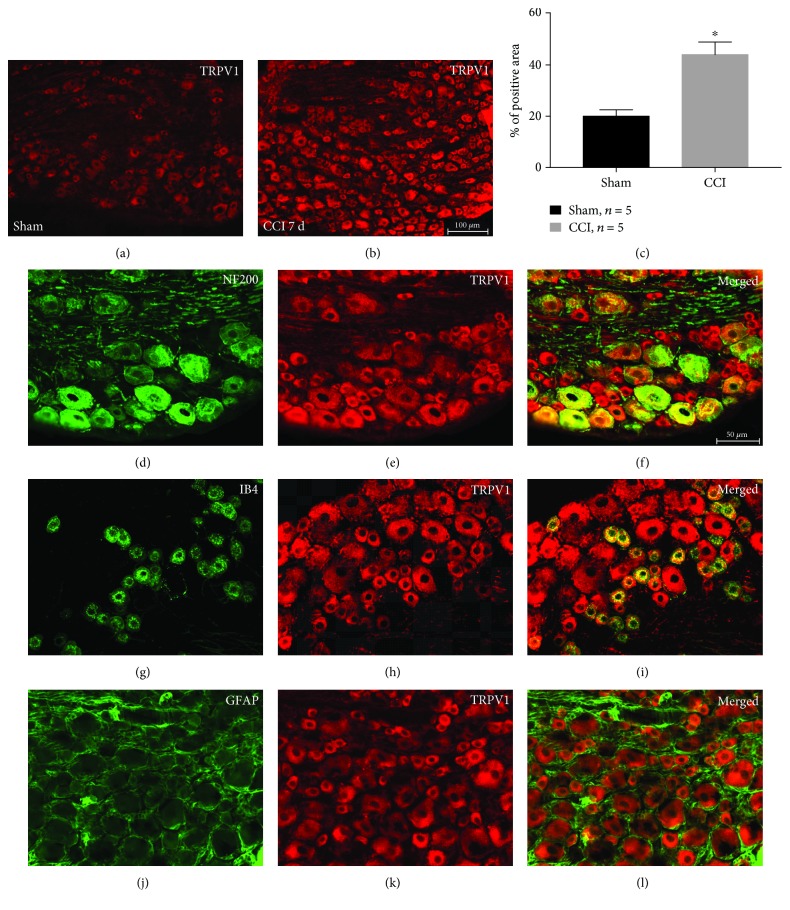
TRPV1 immunoreactivity (IR) in DRGs increases after CCI. (a, b) Representative images showing TRPV1 IR in DRGs for sham-surgery control (a) and at 7 days post-CCI (b). Scale bar for (a) and (b): 100 *μ*m. (c) Quantification of TRPV1-positive area in DRGs. ^∗^*P* < 0.05 vs. the sham group Student's *t*-test. (d–l) Double-labeled immunofluorescent images showing the TRPV1 (red: (e), (h), and (k)) colocalized with NF200 (green: (d)) and IB4 (green: (g)), but not GFAP (green: (j)). The pairs of images are merged in (f), (i), and (l). Scale bar for (d)–(l): 50 *μ*m.

To verify previous results, we used immunohistochemistry to detect the expression and distribution of MZF1 and TRPV1 in DRGs following CCI. As expected, our results revealed that CCI obviously increased the expression of MZF1 (compared with that of the sham group, *P* < 0.05, Student's *t*-test, Figure 2(a)–2(c)) and TRPV1 (*P* < 0.05, Student's *t*-test, Figure 3(a)–3(c)), which was in accordance with our Western blotting results. Double-immunofluorescent staining revealed that, in DRGs, MZF1 mainly coexpressed with NF200 (A-type neuron marker), IB4 (C-type nonpeptidergic neuron marker) and GFAP (satellite glial-cell marker) after CCI (Figure 2(d)–2(l)). In contrast, the augmented TRPV1 only colocalized with NF200 and IB4 (Figure 3(d)–3(l)). This data suggests that MZF1 and TRPV1 may be coexpressed in DRG neurons during neuropathic pain.

### 3.2. Effect of DRG MZF1 Overexpression on Naïve Rats

**Figure 4:**
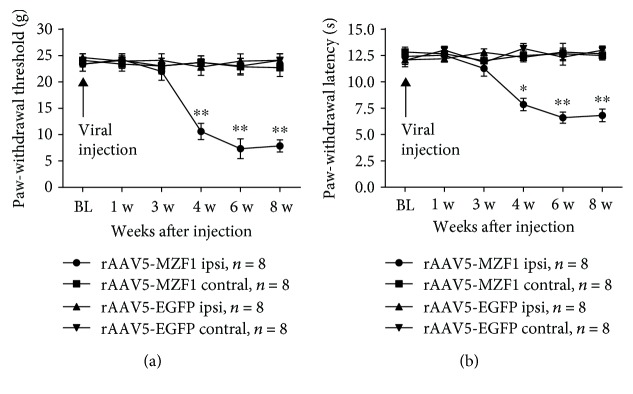
Effect of DRG microinjection of rAAV5-MZF1 on naïve rats. Rats receiving DRG microinjections of rAAV5-MZF1 displayed a significant mechanical allodynia (a) and thermal hyperalgesia (b) on the ipsilateral side of the injection site. ^∗^*P* < 0.05 and ^∗∗^*P* < 0.01 vs. rAAV5-EGFP on the ipsilateral side (two-way ANOVA).

Next, we examined whether overexpression of MZF1 could mimic CCI-induced nociceptive sensitization in naïve rats via microinjection of rAAV5-MZF1 or rAAV5-EGFP into the DRG. Rats injected with rAAV5-MZF1 displayed a significant decrease in PWT (compared with the baseline value, 4 w, *P* < 0.01; 6 w, *P* < 0.01; and 8 w, *P* < 0.01; two-way ANOVA, Figure 4(a)) and PWL (compared with the baseline value, 4 w, *P* < 0.05; 6 w, *P* < 0.01; and 8 w, *P* < 0.01; two-way ANOVA, Figure 4(b)) of the ipsilateral planta pedis compared with those of the rAAV5-EGFP group; a statistical difference occurred at 4 weeks and was maintained at 8 weeks postinjection. Microinjection of rAAV5-MZF1 had no influence in PWL and PWT on the contralateral side (Figures 4(a) and 4(b)).

### 3.3. Effect of DRG MZF1 Knockdown on the Development and Maintenance of Neuropathic Pain Induced by CCI

**Figure 5:**
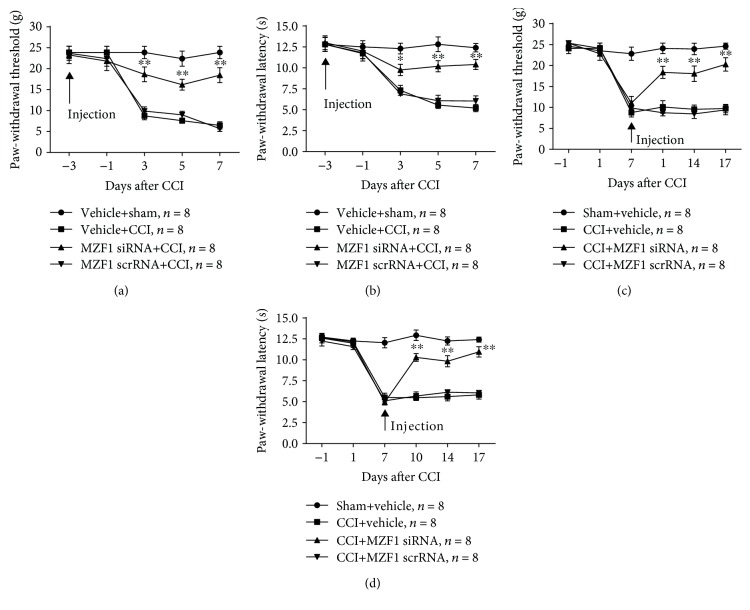
Effect of premicroinjection and postmicroinjection of MZF1 siRNA on CCI-induced neuropathic pain. (a, b) Premicroinjection of MZF1 in the ipsilateral DRG prevented the CCI-induced decrease in PWT (a) and PWL (b). ^∗^*P* < 0.05 and ^∗∗^*P* < 0.01 vs. the vehicle+CCI group (two-way ANOVA). (c, d) Postmicroinjection of MZF1 in the ipsilateral DRG abolished the established CCI-induced nociceptive changes in PWT (c) and PWL (d). ^∗∗^*P* < 0.01 vs. the CCI+vehicle group (two-way ANOVA). No change was observed in PWT and PWL of rats premicroinjected or postmicroinjected with MZF1 scrRNA (a–d).

To further verify the role of MZF1 in DRGs for the development of neuropathic pain in CCI rats, MZF1 siRNA was microinjected into the DRG three days before CCI surgery. Compared with that of the vehicle+CCI group, MZF1 siRNA significantly prevented the CCI-induced reduction in PWT (3 d, *P* < 0.01; 5 d, *P* < 0.01; and 7 d, *P* < 0.01; two-way ANOVA, Figure 5(a)) and PWL (3 d, *P* < 0.05; 5 d, *P* < 0.01; and 7 d, *P* < 0.01; two-way ANOVA, Figure 5(b)) that occurred at 3 d and persisted for at least 7 d post-CCI. Although MZF1 siRNA blocked the development of mechanical allodynia and thermal hyperalgesia in CCI, the role of MZF1 in late-phase neuropathic pain still needed to be examined. For this purpose, we microinjected MZF1 siRNA into the DRG on the seventh day after CCI induction. Subsequently, the results of behavioral testing showed that MZF1 siRNA reversed the CCI-induced decrease in PWT (3 d, *P* < 0.01; 5 d, *P* < 0.01; and 7 d, *P* < 0.01; two-way ANOVA, Figure 5(c)) and PWL (3 d, *P* < 0.01; 5 d, *P* < 0.01; and 7 d, *P* < 0.01; two-way ANOVA, Figure 5(d)) compared with those of the CCI+vehicle group, which occurred at 3 d and persisted for 10 d postinjection. Pretreatment and posttreatment of MZF1 scrRNA had no influence on the PWT or PWL of rats.

### 3.4. Effect of MZF1 Overexpression and Knockdown on the Expression of TRPV1 in DRGs

**Figure 6:**
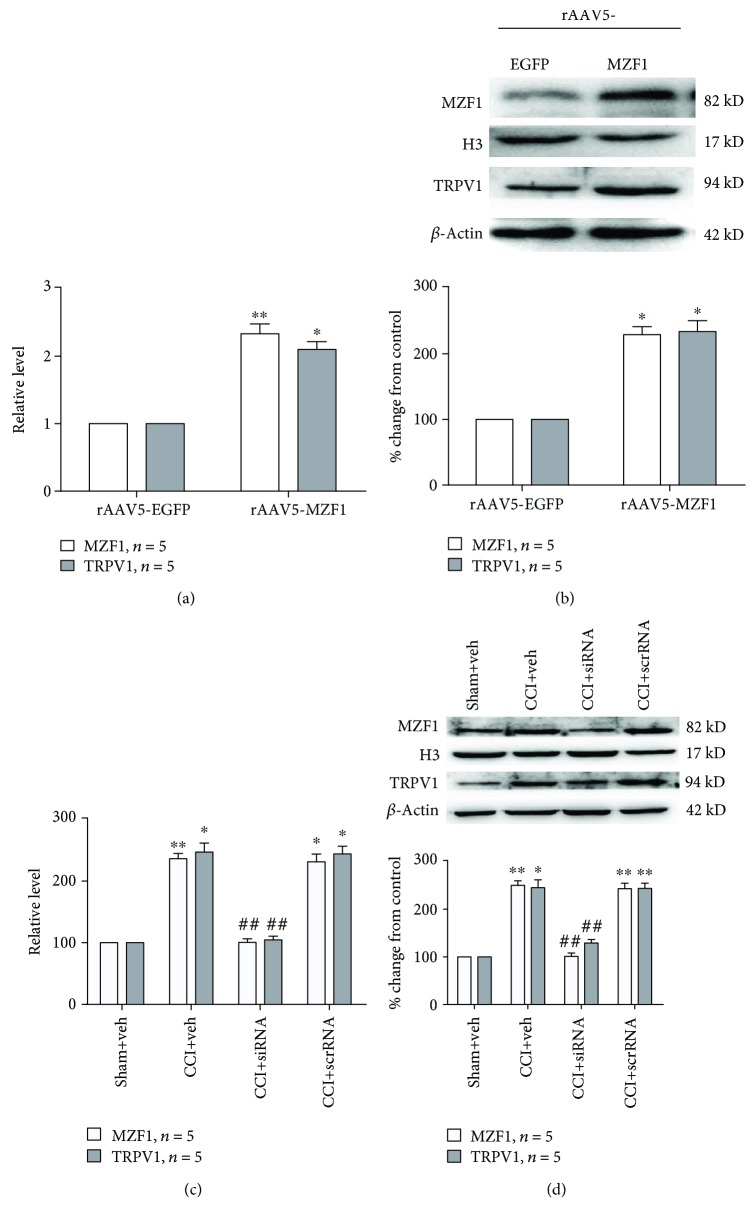
TRPV1-mediated CCI-induced neuropathic pain via regulation by MZF1 in DRGs. (a, b) DRG microinjection of rAAV5-MZF1 increased MZF1 and TRPV1 mRNA levels (a) and protein expression (b). ^∗^*P* < 0.05 and ^∗∗^*P* < 0.01 vs. the rAAV5-EGFP group (Student's *t*-test). (c, d) DRG microinjection of MZF1 siRNA abolished CCI-induced increases in MZF1 and TRVP1 mRNA levels (c) and protein expression (d). ^∗^*P* < 0.05 and ^∗∗^*P* < 0.01 vs. the sham+vehicle group and ^##^*P* < 0.01 vs. the CCI+vehicle group (one-way ANOVA).

Previous studies have shown that MZF1 regulates multiple ion channels, so we assessed whether MZF1 was associated with TRPV1 in naïve and neuropathic pain. The protein and mRNA expressions of TRPV1 in DRGs were examined after rats received microinjections of rAAV5-MZF1. Compared with that of the rAAV5-EGFP group, qRT-PCR revealed that rAAV5-MZF1 significantly increased the mRNA expression of TRPV1 (*P* < 0.05, Student's *t*-test, Figure 6(a)) and MZF1 (*P* < 0.01, Student's *t*-test, Figure 6(a)) in DRGs. The result of Western blotting showed that, compared with that of the rAAV5-EGFP group, rAAV5-MZF1 significantly induced an increased expression of TRPV1 (*P* < 0.05, Student's *t*-test, Figure 6(b)) and MZF1 (*P* < 0.05, Student's *t*-test, Figure 6(b)) protein levels. In order to examine whether increased MZF1 was associated with TRPV1 in CCI-induced neuropathic pain, we used DRG microinjections of MZF1 siRNA to explore the effect of knockdown of MZF1 on expression of TRPV1. Compared with those in the CCI+vehicle group, the quantities measured by qRT-PCR showed that MZF1 siRNA prevented the increase in mRNA expression of TRPV1 (CCI+veh vs. CCI+siRNA, *P* < 0.01, one-way ANOVA, Figure 6(c)) and MZF1 (CCI+veh vs. CCI+siRNA, *P* < 0.01, one-way ANOVA, Figure 6(c)) in DRGs. Similarly, the results of Western blotting showed that MZF1 siRNA significantly reduced the protein expression of TRPV1 (CCI+veh vs. CCI+siRNA, *P* < 0.01, one-way ANOVA, Figure 6(d)) and MZF1 (CCI+veh vs. CCI+siRNA, *P* < 0.01, one-way ANOVA, Figure 6(d)) in DRGs compared with those in the CCI+vehicle group. Compared with that of the CCI+vehicle group, the treatment of MZF1 scrRNA had no influence on either protein or mRNA expression of MZF1 and TRPV1 in DRGs. Since MZF1 and TRPV1 were only coexpressed in DRG neurons, the role of MZF1 in satellite glial cells also needed to be investigated. Recent studies had demonstrated that purinergic P2X7 receptors (P2X7Rs) contribute to chronic pain via mediating ATP and cytokine release from satellite glial cells [24]. Our results also showed that CCI increased the protein expression of P2X7R in DRGs (compared with that of the sham group, 3 d, *P* < 0.01; 7 d, *P* < 0.01; 10 d, *P* < 0.05; and 14 d, *P* < 0.05; one-way ANOVA, Supplementary [Supplementary-material supplementary-material-1]). However, microinjection of MZF1 siRNA did not affect the expression of P2X7R protein levels (Supplementary [Supplementary-material supplementary-material-1]).

### 3.5. Effect of Knockdown of TRPV1 on Development and Maintenance of Neuropathic Pain Induced by CCI

**Figure 7:**
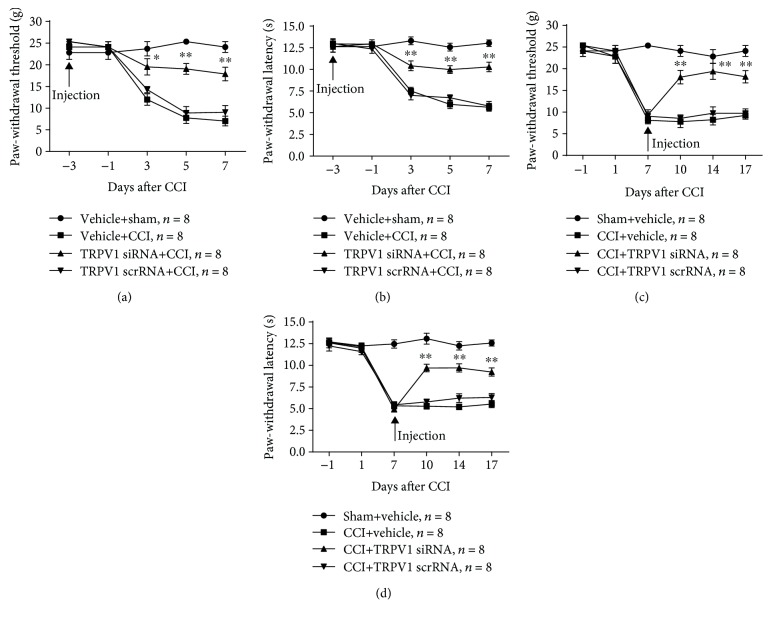
Effect of premicroinjection and postmicroinjection of TRPV1 siRNA in CCI-induced neuropathic pain. (a, b) Premicroinjection of TRPV1 in the ipsilateral DRG prevented CCI-induced decreases in PWT (a) and PWL (b). ^∗^*P* < 0.05 and ^∗∗^*P* < 0.01 vs. the vehicle+CCI group (two-way ANOVA). (c, d) Postmicroinjection of TRPV1 in the ipsilateral DRG abolished the established CCI-induced nociceptive changes in PWT (c) and PWL (d). ^∗∗^*P* < 0.01 vs. the CCI+vehicle group (two-way ANOVA). No change was observed in PWT and PWL of rats premicroinjected or postmicroinjected with MZF1 scrRNA (a–d).

In order to determine the role of TRPV1 in the development of neuropathic pain, TRPV1 siRNA was microinjected in DRGs three days before CCI induction. Compared with that of the vehicle+CCI group, TRPV1 siRNA prevented the CCI-induced decrease in PWT (3 d, *P* < 0.05; 5 d, *P* < 0.01; and 7 d, *P* < 0.01, two-way ANOVA, Figure 7(a)) and PWL (3 d, *P* < 0.01; 5 d, *P* < 0.01; and 7 d, *P* < 0.01; two-way ANOVA, Figure 7(b)), starting at 3 d post-CCI and persisting for at least 7 d post-CCI. Next, to explore the effect of TRPV1 in the maintenance of neuropathic pain, we microinjected TRPV1 siRNA at 7 d post-CCI. Compared with that of the CCI+vehicle group, TRPV1 siRNA reversed the reduction of PWT (3 d, *P* < 0.01; 5 d, *P* < 0.01; and 7 d, *P* < 0.01; two-way ANOVA, Figure 7(c)) and PWL (3 d, *P* < 0.01; 5 d, *P* < 0.01; and 7 d, *P* < 0.01; two-way ANOVA, Figure 7(d)) in CCI rats, starting at 3 d postinjection and persisting for at least 10 d postinjection. Pre- or postmicroinjection of TRPV1 scrRNA had no effect on neuropathic pain induced by CCI.

## 4. Discussion

The CCI model is characterized by allodynia and hyperalgesia, and it imitates clinical neuropathic pain. Here, we demonstrated that MZF1 and TRVP1 are required for the different phases of CCI-induced neuropathic pain and elucidated the relationship between MZF1 and TRPV1 in pathological processes of neuropathic pain. In the present study, we revealed that CCI increased the mRNA and protein expression of MZF1 and TRPV1 in L4/5 DRGs. Overexpression of MZF1 through DRG microinjection of rAAV5-MZF1 reduced the PWT and PWL of naïve rats. Prior microinjection of MZF1 siRNA prevented the development of neuropathic pain. For established neuropathic pain, administration of MZF1 siRNA via DRG microinjection on day 7 after CCI reversed the PWL and PWT reduction induced by CCI. Next, DRG microinjection of rAAV5-MZF1 increased the mRNA level and protein expression of TRPV1. Moreover, there was a CCI-induced increase in both the mRNA level and protein expression of TRPV1 in DRGs, which was inhibited by MZF1 siRNA via DRG microinjection. Pretreatment and posttreatment of TRPV1 siRNA via DRG microinjection blocked the development of CCI-induced neuropathic pain and reversed the mechanical allodynia and thermal hyperalgesia in the maintenance phase of neuropathic pain. Collectively, these results indicate that CCI induces an increase in the expression of MZF1 and TRPV1 in DRGs and that the upregulation of MZF1 may contribute to development and maintenance of neuropathic pain via its mediation of TRPV1.

Although the underlying mechanisms of neuropathic pain are not clearly understood, it is known that peripheral sensitization and central sensitization are important for the development and maintenance of neuropathic pain [25]. DRGs are located in the peripheral nervous system, which contains many types of neurons and glial cells. Substantial evidence has indicated that the maladaptive molecular changes of primary sensory neurons in DRGs result in peripheral sensitization and that stimulation of DRGs decreases hyperexcitability of DRG neurons and reduces nociceptive responses [26, 27]. It has been reported that MZF1 and TRPV1 are increased in DRGs following peripheral nerve trauma [9, 28], but few studies have systematically aimed to characterize temporal profiles and cell distributions of MZF1 and TRPV1 after nerve injury. MZF1 has attracted substantial attention due to its involvement in mediating ion channels. CCI induces an increase in the expression of MZF1 and promotes the binding activity of MZF1 to Kv1.2 antisense RNA, which blocks the expression of the Kv1.2 gene and thus contributes to neuropathic pain [9]. In the present study, we also demonstrated upregulation of the mRNA level and protein expression of MZF1 in DRGs compared with those in the sham group; the statistically significant difference began on day 3 and persisted for more than 14 days after CCI. The transient receptor potential family is one of the largest groups of receptors, where some subtypes function as noxious-stimulus detectors in DRGs. Among these subtypes, many studies have demonstrated that TRPV1 is involved in various pain models [29, 30]. TRPV1 is an integrator of peripheral nociceptive information administered by different forms of stimulants [31]. After partial nerve injury, the expression of TRPV1 increases in both damaged and undamaged DRG neurons [32]. Some K^+^ channels are expressed in the DRG cell membrane, such as Kv; large-conductance, voltage-, and Ca^2+^-activated K^+^ (BK) channels; and the two-pore domain potassium channel TREK1, which form a functional complex with TRPV1. The Ca^2+^ influx caused by TRPV1 activation is an important condition for spontaneous discharge (spontaneous activity, SA) of the DRG small cell [33–35]. TRPV1 activation inhibits the intracellular K^+^ efflux induced by the opening of K^+^ channels via mediation of Ca^2+^, Na^+^, and other cationic influxes, which has a critical effect on the release frequency of spontaneous potential, resting potential, and excitability of DRG neurons [36]. The results of the present study revealed that CCI led to robust increased mRNA and protein levels of TRPV1 in DRGs, beginning at 3 days post-CCI and lasting as long as 14 days post-CCI. The inflammation induced by pain from complete Freund's adjuvant (CFA) significantly increases the expression of TRPV1 and sensitizes TRPV1 in DRG neurons [37], which suggests that TRPV1 is involved in various types of pain; this is consistent with our present results. The changes in expressions of MZF1 and TRPV1 were consistent with behavioral changes following CCI, which lasted for more than two weeks.

A large number of studies have demonstrated that MZF1 and TRPV1 in DRGs are crucial for the pathogenesis of chronic pain [8, 38, 39], but since the pathological processes of chronic pain are separated into early and later phases (i.e., development and maintenance phases), the exact roles of MZF1 and TRPV1 in different phases of chronic pain have not been fully understood. To explore the effect of CCI-induced upregulation of MZF1 in the development and maintenance of neuropathic pain, DRG microinjections of MZF1 siRNA were administered 3 days before and 7 days after CCI induction. The behavioral results showed that prior administration of MZF1 siRNA significantly prevented the CCI-induced mechanical allodynia and thermal hyperalgesia, while post administration of MZF1 siRNA reversed the established neuropathic pain induced by CCI. Next, we microinjected rAAV5-MZF1 into naïve rats to evaluate the effect of overexpression of MZF1 and found that, like CCI-induced nociceptive behavior, naïve rats microinjected with rAAV5-MZF1 displayed a significant reduction in PWT and PWL. This result suggests that MZF1 in DRGs plays a critical role in the induction of neuropathic pain, which corroborates the results of Li and colleagues [9]. Previous studies have demonstrated that knockdown and pharmacological inhibition of TRPV1 relieve neuropathic pain [40–42]. However, these studies have little definitive evidence to support that TRPV1 directly contributes to the genesis of neuropathic pain [43]. In order to investigate the role of TRPV1 in the development and maintenance of neuropathic pain, rats were microinjected with TRPV1 siRNA 3 days before and 7 days after CCI induction. Our results indicated that TRPV1 siRNA dramatically prevented the induction of the CCI-induced decreases in PWT and PWL (when administered 3 days prior to CCI) and alleviated the established neuropathic pain (when administered 7 days post-CCI). These behavioral results demonstrated that the upregulation of TRPV1 contributes to the development and maintenance of neuropathic pain.

TRPV1, which is an important nonselective ion channel, has been associated with peripheral sensitization and central sensitization. It has been reported that expression and differentiation of TRPV1 are regulated by specific transcription factors, such as Runx1, Sp1, Sp4, and Klf7 [44–46]. Klf7 is a Kruppel-like zinc-finger transcription factor which is similar to MZF1. Li and colleagues have demonstrated that MZF1 contributed to neuropathic pain via regulating the Kv1.2 channel. Similar to previous studies, our results also showed augmented expression of MZF1 and TRPV1 in DRGs following induction of neuropathic pain. However, the mechanism of MZF1 and the interaction between MZF1 and TRPV1 in neuropathic pain have not been fully understood. In the present study, we found that the increased expression of MZF1 paralleled the increased expression of TRPV1 in DRGs following CCI. DRG microinjection of rAAV5-MZF1 robustly increased mRNA and protein levels of MZF1 and TRPV1 in DRGs, which suggests that MZF1 may promote the transcription of TRPV1 in DRGs of naïve rats. In the present study, we also observed that CCI induced a lasting upregulation of TRPV1. However, this upregulation was inhibited by DRG microinjection of MZF1 siRNA. These results indicate that MZF1 is associated with CCI-induced increases in TRPV1. Double-immunofluorescent labeling showed that the increased MZF1 localized in DRG neurons and satellite glial cells but the increased TRPV1 was only localized in DRG neurons. These findings imply that CCI-induced upregulation of MZF1 and TRPV1 may colocalize in DRG neurons. Our present data suggests that CCI-induced upregulation of MZF1 may participate in the development and maintenance of neuropathic pain through mediation of one of its key downstream targets, TRPV1, in DRG neurons. However, the role of MZF1 in satellite glial cells remains unclear. A previous study has shown that P2X7R is involved in neuronal soma and satellite glial-cell communication in sensory ganglia [24]. In the present study, the data from Western blotting demonstrated that CCI increased the protein expression of P2X7R in DRGs, beginning at 3 days post-CCI and lasting for at least 14 days post-CCI. DRG microinjection of MZF1 siRNA did not change CCI-induced increases of P2X7R protein levels. Therefore, MZF1 in satellite glial cells may contribute to neuropathic pain via other signaling pathways, which deserves further investigation.

In summary, our results demonstrate that CCI results in an increase in the expression of MZF1 and TRPV1 in DRGs. Inhibiting the expression of MZF1 or TRPV1, before or after CCI induction, prevents the initiation of neuropathic pain and alleviates established neuropathic pain, respectively. The increased MZF1 contributes to the development and maintenance of neuropathic pain via TRPV1. Therefore, the MZF1/TRPV1 signaling pathway may be a potential therapeutic target for preventing and resolving neuropathic pain.

## Figures and Tables

**Table 1:** Primers used for qRT-PCR.

Primer names	Sequences
MZF1-F	AAG TAG AAG GCA TCT TGT CGC
MZF1-R	CCT GGA TCG CTG GGG AGT
TRPV1-F	TTC CGA GGG ATT CAG TAT TT
TRPV1-R	TGA GCA GGA GGA TGT AGG TG
GAPDH-F	CACGAATTCGGTCATCATCTCTGCCCCCTCTGC
GAPDH-R	GCTGGATCCGACGCCTGCTTCACCACCTTCTT

RT: reverse transcription; F: forward; R: reverse.

## Data Availability

The data used to support the findings of this study are available from the corresponding author upon request.

## Abbreviations

MZF1: Myeloid zinc finger 1
Klf7: Kruppel-like zinc-finger transcription factor 7
TRPV1: Transient receptor potential vanilloid 1
P2X7R: Purinergic P2X7 receptor
CCI: Chronic-constriction injury
DRG: Dorsal root ganglion
PWT: Paw-withdrawal threshold
PWL: Paw-withdrawal latency.

## Supplementary Materials

Supplementary Materials The concise description has been stated under Results (Section 3.4 Effect of MZF1 Overexpression and Knockdown on the Expression of TRPV1 in DRGs). Supplementary Figure 1: (A) Western blotting data showing an increase in protein expression of P2X7R in L4/5 DRGs following CCI. ^∗^*P* < 0.05 and ^∗∗^*P* < 0.01 vs. the sham group (one-way ANOVA). (B) DRG microinjection of MZF1 siRNA did not affect CCI-induced increase in P2X7R protein expression. ^∗∗^*P* < 0.01 vs. the sham+veh group (one-way ANOVA).
