# Supplementary figures and images for: MZF1 in the Dorsal Root Ganglia Contributes to the Development and Maintenance of Neuropathic Pain via Regulation of TRPV1
Source: Neural Plast. 2019 Sep 8;2019:2782417. doi: 10.1155/2019/2782417 (PMC6754943; doi:10.1155/2019/2782417)

Supplementary Figure 1.


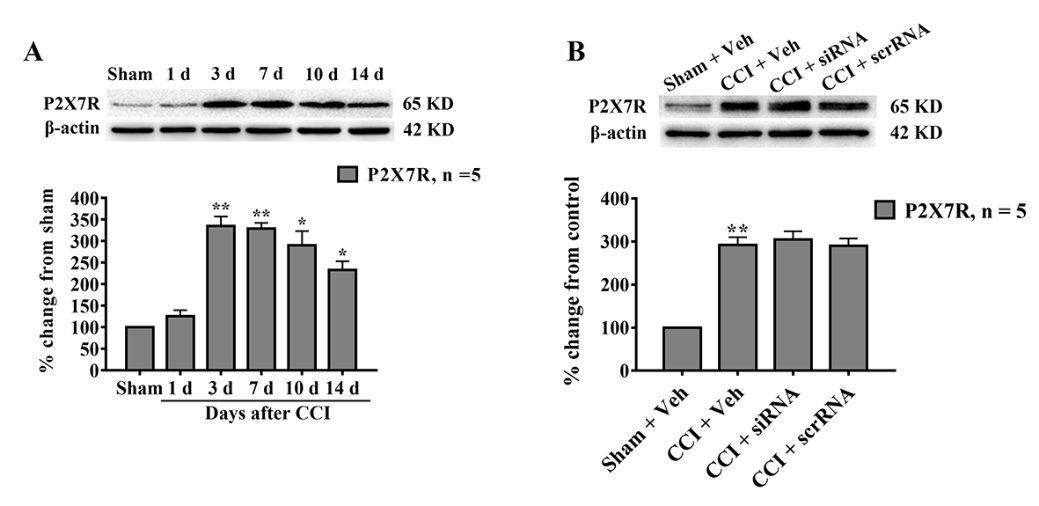

Supplement: Supplementary Materials — The concise description has been stated under Results (Section 3.4 Effect of MZF1 Overexpression and Knockdown on the Expression of TRPV1 in DRGs). Supplementary Figure 1: (A) Western blotting data showing an increase in protein expression of P2X7R in L4/5 DRGs following CCI. ∗P < 0.05 and ∗∗P < 0.01 vs. the sham group (one-way ANOVA). (B) DRG microinjection of MZF1 siRNA did not affect CCI-induced increase in P2X7R protein expression. ∗∗P < 0.01 vs. the sham+veh group (one-way ANOVA). [file 2782417.f1.docx]
